# Is Population Density Associated with Non-Communicable Disease in Western Developed Countries? A Systematic Review

**DOI:** 10.3390/ijerph19052638

**Published:** 2022-02-24

**Authors:** Elaine Ruth Carnegie, Greig Inglis, Annie Taylor, Anna Bak-Klimek, Ogochukwu Okoye

**Affiliations:** 1 School of Health and Social Care, Edinburgh Napier University, Sighthill Court, Edinburgh EH114BN, UK; annie.taylor@napier.ac.uk (A.T.); a.bak-klimek@napier.ac.uk (A.B.-K.); ogochukwu.okoye@napier.ac.uk (O.O.); 2 School of Education and Social Sciences, Paisley Campus, University of the West of Scotland, Paisley PA12BE, UK; greig.inglis@uws.ac.uk

**Keywords:** population density, environmental health, non-communicable disease, health inequalities, built environment and social environments, urban, rural environments, environmental pollution

## Abstract

Over the last three decades, researchers have investigated population density and health outcomes at differing scale. There has not been a systematic review conducted in order to synthesise this evidence. Following the Preferred Reporting Items for Systematic Reviews (PRISMA) guidelines, we systematically reviewed quantitative evidence published since 1990 on population density and non-communicable disease (NCD) within Westernised countries. Fifty-four studies met the inclusion criteria and were evaluated utilising a quality assessment tool for ecological studies. High population density appears to be associated with higher mortality rates of a range of cancers, cardiovascular disease and COPD, and a higher incidence of a range of cancers, asthma and club foot. In contrast, diabetes incidence was found to be associated with low population density. High and low population density are therefore risk markers for a range of NCDs, indicating that there are unidentified factors and mechanisms underlying aetiology. On closer examination, our synthesis revealed important and complex relationships between population density, the built environment, the nature of greenspace and man-made exposures. In light of increasing rates of morbidity and mortality, future research is required to investigate these associations in order to establish causative agents for each NCD.

## 1. Introduction

The World Health Organisation (WHO) has estimated that 38 million deaths per annum are caused by non-communicable diseases (NCDs) such as heart disease, stroke, cancer, diabetes and chronic lung disease [1]. The growing prevalence of NCDs has been attributed to factors such as an ageing population, the emergence of advanced medical technologies and environmental and lifestyle factors [2]. Individual and collective behaviours appear to lead to the interaction of genetic, environmental and infrastructure risk factors [3]. Spatial planning decisions and the design of the built environment shape human activity within a specific bounded landscape [4], leading to human habitats becoming potentially obesogenic and diabetogenic [5]. In contrast and outside of an individual’s control, exposure to emissions from man-made activities can lead to cardiovascular and oncological outcomes [6]. For example, economic activity leads to air pollution and subsequent exposure to local residents. The ingestion and inhalation of fine particles poses a risk to their health through a combination of social, environmental and genetic risk [7]. Whether populations benefit or are disadvantaged by these interactions will depend on the nature of these activities, the spatial dimension—how compact or diffuse the built environment/neighbourhood is (areal density)—and the temporal dimension—the number of people residing/working/visiting the area at any given time (residential and population density) [8].

There is divergent evidence when comparing the effects of urbanicity and rurality on the development of disease [9,10,11]. For a systematic review, it is imperative to clearly define population density and explain how it is measured. Population density is a geographical term denoting the number of persons in a specified area of land. However, in some studies population density is poorly defined or limited to population size, indicating measures of rurality rather than the number of residents per square kilometre [12,13]. Precision public health analysis requires the accurate mapping of population density when combining with epidemiological and exposure data [14].

Both density of population and proximity to man-made activities, as well as the natural environment, concentrate risk for humans [15]. Population density is therefore a measure of “the degree of pressure on natural resources” as well as an indicator reflecting the level of human activity within a specific bounded landscape [4] (p. 69). Exposure to contaminants from soil, water or air coupled with social or economic vulnerability and population density may increase potential harm to health at a subclinical or clinical level. Therefore, mitigating actions to improve environmental quality may lead to potential health and economic savings with the greatest improvement being seen within areas of both high density and high human activity [16]. The public have a right to know about their contexts and implications of living within that context for health outcomes. Martenies et al. (2017) emphasise the importance of this right, stating that “attributable health burdens are a function of exposures, susceptibility and vulnerability (e.g., baseline incidence rates), and population density” [17] (p. 1).

Relative population density varies within countries, regions and continents. Asian and European cities are more densely populated than those in the United States (US) [18]. Comparisons across continents with differing levels of development will limit an examination of core factors that may be associated with the development of NCDs. It will therefore be necessary to examine continents and countries which share similar economic, political, cultural and health systems such as high-income countries within the Western Hemisphere. It is important to delineate the boundaries of a neighbourhood or region as size will influence the strength and direction of observed relationships between variables [19]. There are advantages to investigating health effects across large areas of land in order to adequately capture the aetiology and distribution of disease as well as examining outcomes at neighbourhood level and spatial mapping in order to identify exposure gradients [17]. For example, positive trends were observed between population density and annual cancer incidence across a quarter of US counties [20], and population density and exposure to traffic-related air pollution across seven world cities [18]. Population density is a potential risk marker as it signifies where the highest number of people may be exposed and also the highest number from particular vulnerable groups. This review focused on the state of knowledge regarding population density and the development of antecedents of disease within developed Western nations.

Population density as a variable can serve as a proxy or surrogate for any number of explanatory variables that lead to the development of a specific disease or mechanisms. It is important to focus on and examine each variable that may be causing an effect, such as the built environment; the physical and social environment; the quality of neighbourhoods (including mobility rates and overcrowding); as well as which population groups are impacted, whether by age, sex, ethnicity, or other group characteristics. In a systematic review it will be important to define and investigate other potentially significant confounders such as area-level and individual-level socio-economic status which may influence outcomes in one particular space [21].

The objective of this review was to obtain a comprehensive overview of existing empirical evidence on the relationship between population density and NCDs. This included investigating the extent to which empirical evidence supports any relationship between population density and the development of non-communicable disease outcomes within Western countries (and whether this relationship is independent of socioeconomic status). To address this, three specific research questions were developed:

1. Does the mortality rate of residents in Western developed countries differ by degree of population density?
2. Does the morbidity rate of residents in Western developed countries differ by degree of population density?
3. To what degree do socioeconomic determinants explain any differences in morbidity or mortality rates?

## 2. Materials and Methods

The review was conducted according to the Preferred Reporting Items for Systematic Reviews and Meta-Analysis (PRISMA) guidelines [22], and the protocol was registered on PROSPERO [23].

### 2.1. Search Strategy

The development of the search strategy was derived from an initial review of the literature in 2018, conducted in June 2019 and updated in June 2020 (Table S1). Owing to an underdevelopment of evidence in this topic area, studies were included if they were published between January 1990 and June 2020. Ten interdisciplinary databases were searched. All studies had to be published in peer reviewed journals, available in the English language, focus on physical health (non-communicable disease) rather than mental health outcomes, and be conducted in Westernised developed countries. Study designs for inclusion were ecological population-based studies: retrospective, longitudinal, epidemiological with ecological design; ecological studies using temporal or spatiotemporal design; or multi-level studies incorporating group and individual level data. In addition, the reference lists of all the studies identified by the search strategy were checked for potentially eligible studies by one author (E.C.). Only studies that met the inclusion criteria and included population density as an independent or covariable were included in the review. The calculation of population density had to be explicit within the study methods for inclusion in this review. All studies that did not meet these criteria were excluded. All references were organised in Endnote. All studies meeting the inclusion criteria (*n* = 221) were independently assessed by two members of the research team, which included reading the full text (E.C. and G.I.). Where there was disagreement between two reviewers, a third member of the research team was asked to review the study (O.O.). Agreement between the two reviewers was 96%, and following the review of the remaining 4% of studies by O.O., 100% consensus was reached.

### 2.2. Quality Assessment

The degree of verification (uncertainty) of any findings from ecological studies continues to be of concern. Methodological challenges include “lack of variation in the ecological exposure (and health outcome) in the available data; selection bias; confounding at both the ecological and individual level; misclassification of variables, units of analysis and assignment of individuals to those units; and multicollinearity” [24] (p. 367). There are no validated tools to assess the quality of ecological studies. As mentioned above, methodological concerns regarding the rigour of ecological studies include risk of ecological bias (attributing group level effects to individual effects), and choice of unit of analysis (how the data have been aggregated at scale or boundary level) [25]. In comparison to individual level studies, ecological studies can utilise a large number of data measurements, demonstrate lower statistical uncertainty for example in mortality rates for a region, access data for a large number of confounders, and the population can be very large and diverse [26]. The STROBE checklist was developed for epidemiologists; however, it does not focus on potential ecological bias nor other forms of bias within spatial analysis [27]. Dufault and Klar (2011) made recommendations for additions to be added to STROBE in order to address these points and standardise reporting [27]. In order to adequately attend to potential ecological biases within selected studies, we adopted a modified version of this scale [25]. Under three headings of “study design”, “statistical methodology” and “quality of reporting”, this scale enables researchers to consider 10 items with a maximum overall score of 12 points. Utilising the study criteria, each item could be scored from 0–2. Studies received an overall grade based on totality of the scores: low (<5 points); medium (5–8 points) and high relevance (>8 points).

## 3. Results

**Figure 1:**
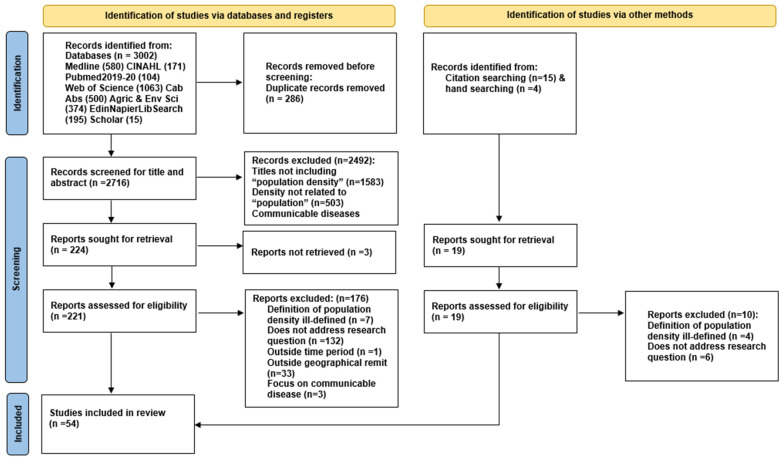
PRISMA 2020 flow diagram for new systematic reviews.

Fifty-four studies were identified for inclusion [3,4,6,11,28,29,30,31,32,33,34,35,36,37,38,39,40,41,42,43,44,45,46,47,48,49,50,51,52,53,54,55,56,57,58,59,60,61,62,63,64,65,66,67,68,69,70,71,72,73,74,75,76,77] (Figure 1).

### 3.1. Data Extraction

Extracted data included country, study design, study population, sampling method, sample size, unit of analysis, outcome measure, statistical methods, main results, confounding factors, co-variables of adjustment and interactions evaluated. Data extraction of the included studies was conducted by one reviewer (E.C., G.I. or O.O.) and checked by a second reviewer (E.C., G.I. or O.O.) (Supplementary File 1).

### 3.2. Study Characteristics

Publication dates ranged from 1991 to 2020 (Table S2). Four studies were published before 2000; sixteen studies were published between 2001 and 2010 and thirty-four studies were published between 2011 and 2021. Most studies were conducted in Europe: in The Netherlands (*n* = 2), Italy (*n* = 1), Ireland (*n* = 3), Sweden (*n* = 5), Poland (*n* = 1), France (*n* = 1), Germany (*n* = 1), Greece (*n* = 1), UK, (*n* = 11), Nordic countries (*n* = 4) and Spain (*n* = 1). Nineteen (*n* = 19) studies were conducted in the USA and four (*n* = 4) were conducted in Australia. Geographical scale varied from four countries (*n* = 1), a single country (*n* = 19), a geographical island (two countries) (*n* = 1), states (*n* = 13), regions (*n* = 9), counties (*n* = 7). One study limited their scope to a single city.

## 4. Quality Assessment

Twenty-eight (52%) of the studies scored high, 25 (46%) scored medium and 1 study scored low (2%) in the assessment scale (Table S3). All studies were included in the analysis (Table S4).

## 5. Associations between Population Density and Health Outcomes

### 5.1. Owing to Heterogeneity of Study Designs, We Used an Aggregative Approach to Provide a Narrative Overview of Existing Empirical Evidence. Studies Were Categorised into the Following Themes for Analysis

1. All-cause mortality
2. Cause-specific mortality:
  - cancer (all cancer combined, all child cancer, and 15 different types of cancer)
  - respiratory (lung cancer, COPD, broad category lung disease/respiratory)
  - cardiovascular (stroke, heart disease)
3. Morbidity
  - cancer (all cancer combined, all child cancer, and 17 different types of cancer)
  - diabetes (type 1 and type 2)
  - respiratory (lung cancer, asthma, broad category lung disease/respiratory)
  - cardiovascular
  - neurological
  - congenital

### 5.2. Key Findings

**Figure 2:**
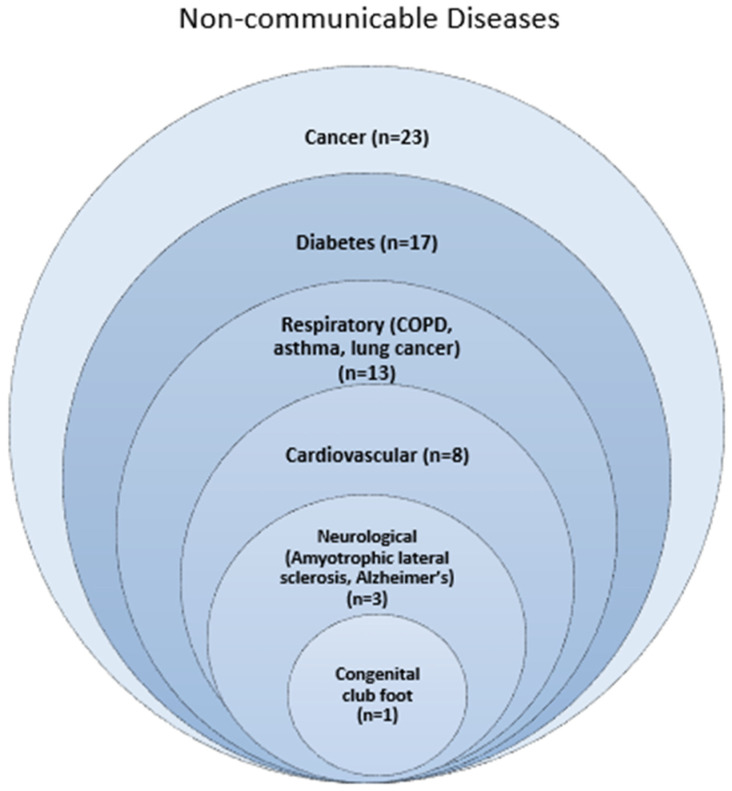
Number of studies focusing on each non-communicable disease.

The majority of studies investigating a relationship between population density focused on cancer mortality and incidence. The majority of non-cancer studies investigated a relationship between population density and diabetes. Other disease groups included respiratory, cardiovascular, neurological and congenital (Figure 2). The NCDs that were most consistently associated with high population density and mortality were a range of cancers, cardiovascular disease and COPD. The NCDs that were most consistently associated with high population density and morbidity were a range of cancers, club foot and asthma.

#### 5.2.1. All-Cause Mortality

**Figure 3:**
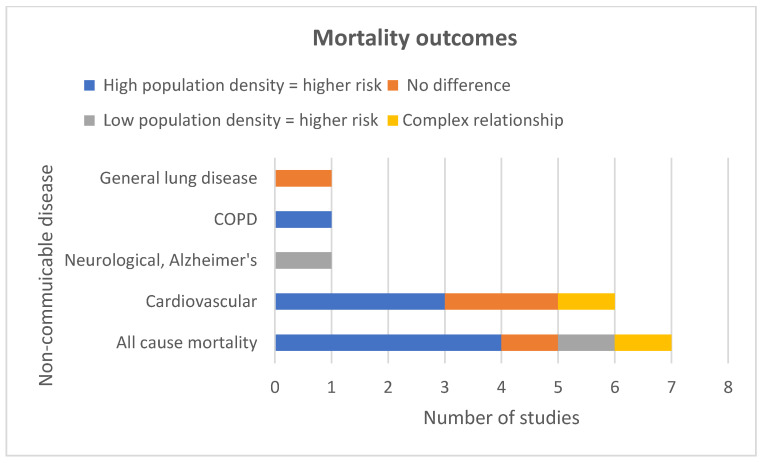
Mortality study outcomes.

**Figure 4:**
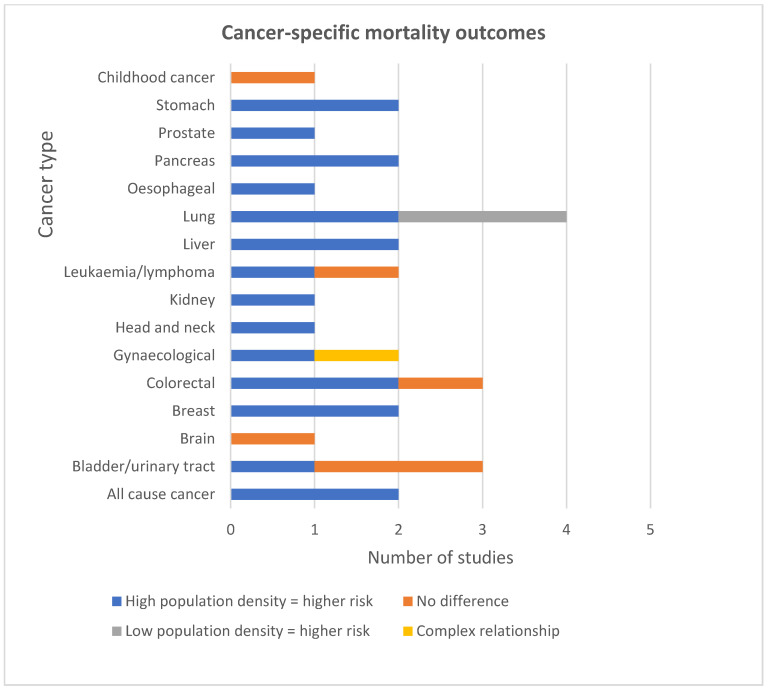
Cancer-specific mortality outcomes.

All-cause mortality was also associated with high population density (Figure 3). Spanning Europe, the United Kingdom and the USA, five of seven all-cause mortality studies reported a consistent pattern of NCDs being associated with high population density [32,44,46,59,73] (Figure 4). A high-quality multilevel study investigated simultaneous effects of area level socioeconomic status (ALSES) and population density on mortality over a 10-year period [59]. They found that there was no gradient for the population density effect. Concentration of risk was only in the most populated areas. However, in the over-50 age group, the proportion of unemployed reduced the effect of population density. In contrast, across all levels of deprivation for British constituencies there was a positive correlation observed between population density and mortality rates, except in the 7th–10th most deprived deciles where there was a negative correlation [73]. Against the overall positive trend, one study found no association with high population density [76], while another described an inverse association, with ‘rural’ areas having higher excess mortality [62]. The authors of this US study suggested that the higher mortality rates in low population density areas could be explained by urban areas having better access to resources. All studies adjusted for socioeconomic status.

#### 5.2.2. Cause Specific Mortality

The conditions that were most consistently associated with high population density and mortality were colorectal, gynaecological, breast, stomach, liver, oesophageal, pancreatic, head and neck, kidney cancers, all-cause cancer, COPD and cardiovascular disease (Figure 3 and Figure 4). The NCDs that were not consistently associated with high population density were Alzheimer’s, testicular cancer, lung cancer, brain cancer, bladder cancer, leukaemia and general lung disease. Findings were from 14 mortality studies, indicating an association between high population density and a range of NCDs [4,6,29,32,33,35,36,38,39,46,47,54,58,63]. Findings for all the specific types of cancer were extracted from seven studies—five high-quality and two medium-quality [4,6,33,35,39,54,58]. Three adjusted for socioeconomic status. For some cancers, mortality was only associated with a specific sex. For example, one study (high-quality) from the USA reported a significant linear relationship between increasing population density and deaths for cancers of the oral cavity and pharynx and oesophagus in men and for liver cancer in women [54]. The same study reported a significant association between higher population density and higher rates of death from head and neck, kidney and pancreatic cancer in men only. For women, the same study revealed a U-shaped pattern of mortality across population quintiles for cervical and rectal cancer [54]. Explanations could include metastasis from cervical to rectal sites related to delay in diagnosis, and/or level of access to health services in these geographical areas. Another high-quality study reported an association between SES and Ischaemic Heart Disease (IHD); mortality was much stronger in densely populated areas, with the 50–64-year-old age group being most affected [36].

#### 5.2.3. Morbidity

**Figure 5:**
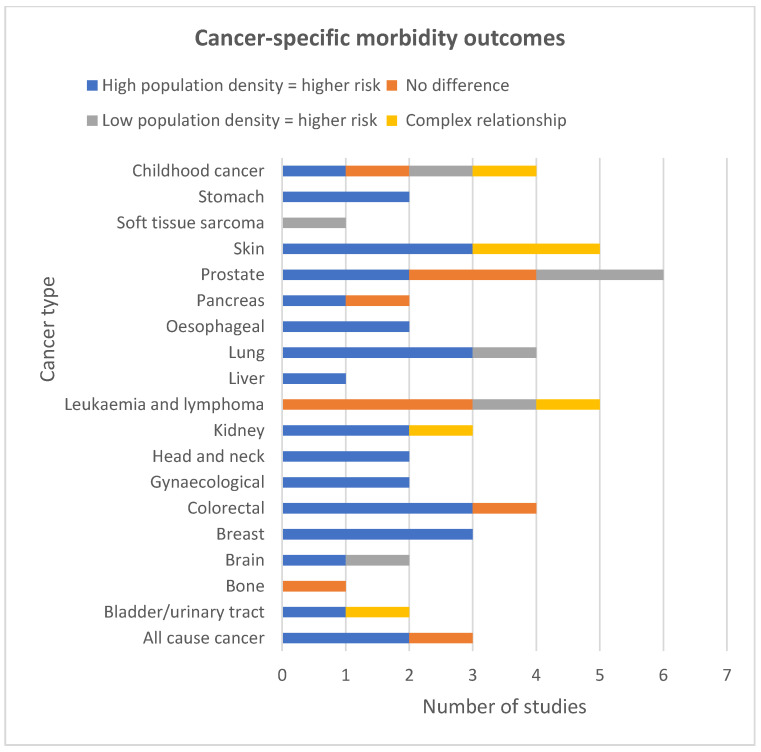
Cancer-specific morbidity study outcomes.

**Figure 6:**
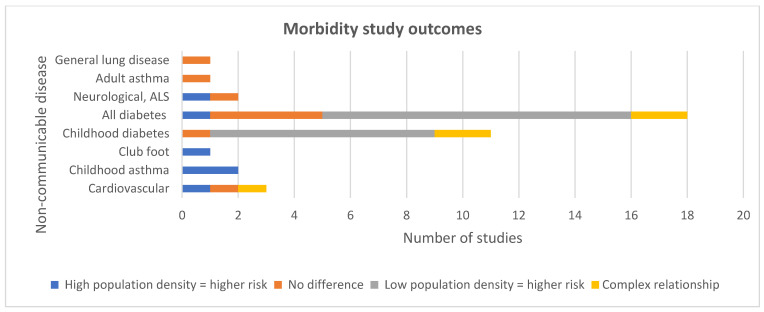
Morbidity study outcomes.

The NCDs that were most consistently associated with high population density and morbidity were lung, colorectal, gynaecological, breast, stomach, liver, oesophageal, head and neck, bladder, kidney, skin cancers, all-cause cancer, club foot and asthma (Figure 5 and Figure 6).

##### Cancers

Three studies consistently reported an association with high population density and high rates of breast cancer [50,60,70] (Figure 5). One study reported a significant linear association between liver cancer and increasing population density in women but not in men [54]. Another study reported a monotonic association between high population density and oral cavity cancer, but only in white women [50]. They found a significant non-monotonic association between population density and oral cavity cancer in black men and white men, with the highest risk of oral cavity cancer in the most densely populated quintile. They did not adjust for SES. Another study that adjusted for SES reported a significant association between high population density and increased rates of head and neck cancer in both men and women [70]. One study reported a significant monotonic association in white men and women and a non-monotonic association between population density and increased risk of stomach cancer in black men, and no association in women [50].

From the USA, Ireland and Denmark, three of four studies investigating lung cancer found an association between high population density and lung cancer incidence [50,60,70]; however, in contrast, one study from France reported that incidence was higher in areas of low population density, and in men [38]. Five studies reported on skin cancer. A significant risk for both sexes of non-melanoma was found in urban areas for men and women [70]. A high-quality study found that, after adjusting for ALSES, risk of basal cell skin cancer was associated with high population density in areas of least deprivation for both sexes. This differed for squamous cell where there was no significant relationship with population density for men [34]. A medium-quality study found that incidence of melanoma was associated with living in areas of high population density and in areas of high SES. However, tumours were diagnosed at an earlier stage in individuals living in areas of high SES rather than those from low SES areas [75]. In contrast, two studies observed an inverted V shaped pattern across population density quintiles for malignant melanoma [50,54]. One of these studies found that this association was only statistically significant in white men [50].

##### Asthma

Two childhood studies reported on whether there was an association between population density and asthma after adjusting for SES [31,53]. One study found that as well as the association with high population density, asthma admission-rate variation was strongly associated with underlying differences in neighbourhood-level characteristics [31]. Neighbourhoods with the highest admission rates had the lowest neighbourhood SES and an aggregated household income close to the federal poverty line. An urban study utilised census data to measure ALSES, ethnicity, population density, proximity to pollution sources and street tree density [53]. They reported that, as well as the association with high population density, street tree density was highest in the most densely populated areas and was associated with a lower prevalence of childhood asthma. The only adult study found no association between population density and asthma incidence, but reported a significant association between income, unemployment and asthma, with those on low incomes and unemployed at a higher risk of developing asthma [77]. Adjusting for age and sex, they found that asthma risk increased with age.

##### Club foot

One study considered population density and club foot [51]. They found a significant positive correlation between club foot and population density. Both club foot and population density had increased across Denmark over the course of the data they included in the study (16 years). Sex was also a risk marker for club foot, with boys being significantly more likely than girls to develop club foot. Potential confounding factors such as individual or ALSES affecting parental health were not included in the analysis.

##### Diabetes

As shown in Figure 6, in contrast to the above trends, only one study reported a correlation between diabetes incidence and high population density (48). Eleven studies reported that people living in areas with low population density were more likely to be diagnosed with type 1 diabetes (T1D) [11,41,42,49,52,55,62,65,67,71,72]. Ten of these 11 studies had adjusted for SES, suggesting that low population density could be a useful marker for risk of type 1 diabetes. However, an Australian high-quality study using generalised additive models observed that although increasing levels of ambient UVR led to decreasing incidence of T1D, in areas of high population density an inverse association was observed [43]. The relationship became null prior to increasing, with rising levels of UVR. The authors surmised that this may be due to shading in urban areas leading to reduced UVR exposure and a lower production of vitamin D. In contrast, another study calculated population weighted daily mean sunshine and annual mean UVR over 25 years across England [71]. Neither were associated with T1D incidence.

Having adjusted for physical activity, a longitudinal study of diabetes risk markers observed a decrease in post-challenge-glucose in adults, aged 45 and over, by 3% in high density and low SES neighbourhoods. In medium and high SES neighbourhoods, blood glucose remained stable or with no change, indicating that high population density may be of greatest advantage for low SES individuals [74].

From the Northern hemisphere, a study conducted at county-level measured age, sex and ALSES indicators annually over a 13-year period [11]. Areas of low population density and high green index were associated with T1D incidence. The green index included forests and grasslands and was thus differing from the definition of urban greenspace. Another state-level study found that counties with higher percentage of rural areas were associated with poorer health outcomes, including obesity, diabetes, premature mortality, and physical inactivity [62]. Counties with more grassland were associated with obesity and physical inactivity but lower levels of self-rated mental and physical distress. Associations were smaller with population density than with their rural indicator. Findings from an urban study revealed an association between T1D and low ALSES, but not with population density [45]. The proportion of green space did not attenuate this finding, suggesting that ALSES may influence health behaviours rather than the availability of “urban” green space.

A high-quality UK study measured spatial, temporal and time interaction effects, comparing incidence of childhood T1D and population density at small area level [55]. Incidence rates increased over time. Lower incidence rates were associated with more densely populated areas, deprivation and population mixing.

## 6. Discussion

Findings from this inaugural systematic review suggest that mortality and morbidity rates of residents in Western developed countries differ by degree of population density for a range of NCDs. In answer to our three research questions, high population density appears to be associated with higher mortality rates of a range of cancers, cardiovascular disease and COPD. Similarly, high population density appears to be associated with a higher incidence of a range of cancers, asthma and club foot. Findings from studies that adjusted for SES indicate that high population density appears to be associated with higher rates of all-cause mortality, cardiovascular disease and COPD mortality as well as incidence of childhood asthma. In contrast, diabetes incidence was found to be associated with low population density.

At the outset of this review, we questioned whether population density would be independently associated with NCDs. Almost fifty years ago, Factor and Waldron (1973) had concluded that high mortality rates are not associated with high population density when socioeconomic status is controlled for [78], and from a planning perspective, Kyrmeyer (1987) had concluded from a literature review that the health effects of density were inconclusive [79]. We reviewed research articles published from 1990 in order to ascertain the state of knowledge on this topic. We expected that high population density would tend to concentrate risk owing to spatial patterning of economic production and subsequent air pollution often found within cities [16]. Although people from a range of socioeconomic groups live in densely populated areas, we suspected that those on lower incomes would have less resources and opportunities to focus on their health and as a result become vulnerable to developing NCDs. They may also be more likely to be exposed to short- and long-term industrial, transport or agricultural emissions. We were not surprised to find that the NCD to receive most attention was cancer. High population density was associated with many types of cancers; however, there are outstanding questions as to why risk varies according to sex for some cancers. It was surprising to observe that cardiovascular conditions were consistently associated with population density and mortality but not morbidity given the range of exogenous and endogenous stressors within built environments.

This study builds on previous evidence and confirms that high- and low-population density are risk markers for a range of NCDs. Causation has already been established for some phenotypes of breast, liver, lung, colon cancer, etc. however, this review highlights that there may be other unidentified factors and mechanisms underlying aetiology [4]. Linear and non-linear trends indicate that there are “particular” factors within specific contexts. For example, findings from two studies [50,54] suggested an inverted ‘v’ shape association between population density and melanoma, with those in quintile 3 being at highest risk.

Childhood cancers were not consistently associated with high population density. This was not surprising owing to the rarity and difficulty in diagnosing childhood cancers; however, childhood asthma and clubfoot were associated with high population density, suggesting that exogenous factors rather than human behaviour may be influencing morbidity rates [31,51,53]. We were particularly interested to note the middle age groups who appeared to be at risk of CHD mortality by living in areas of low ALSES and high population density [36]. In contrast, adults, aged 45 and over benefited from living in densely populated areas in another longitudinal study in relation to blood glucose control [74]. This finding mirrors a recent study set in one English city. Dennis et al. (2020) conducted a spatial analysis investigating the impact of green infrastructure on health outcomes [80], finding that people aged ≥ 60 years were the only age group, living in low-income areas, to gain health benefit from proximity to green space.

Similarly to cancer, across the globe, incidence of T1D has risen dramatically over several decades—3% per annum [81]. We were surprised that the second highest NCD receiving attention from researchers was diabetes and, in particular, childhood diabetes. Our finding that diabetes is associated with low population density reflects previous studies reporting rural/urban variation [82]. A recent spatial analysis investigating T1D incidence in Utah found that T1D decreased with increasing population density [81]. What was most interesting was that 86% of the high-risk clusters were situated in urban areas thus highlighting groups at risk in urban rather than rural areas of low population. It is well known that air pollution blocks UVR; however, it was surprising to find the effect of the built environment forming a canopy and reducing UVR in highly populated areas, leading to a rise in T1D in the context of one country, Australia [43]. Our findings have implications for public health at a local level, both within the built environment and in rural areas. Shared environmental risk factors across several disease groups identified in this review such as UVR exposure, the production of vitamin D, greenspace and infrastructure, all interact. Incorporating a measure of greenspace or a green index appears to be essential when investigating any relationship between NCDs and population density, as demonstrated by one asthma study set in New York City where tree density was associated with lower asthma prevalence in areas of high population density [53].

This review indicates that, apart from cancer, diabetes and several other NCDs, there has been little attention to the potential effects of population density on the development of many other highly prevalent NCDs. Patient groups in Western nations may ask why other widespread diseases have not yet been investigated in relation to high population density, for example, epilepsy. We are not aware of any previous systematic reviews reporting on population density and health outcomes within the Western Hemisphere. There has been little research conducted on population density and non-communicable disease within the Western Hemisphere over the last five years, and this review remains unique in its focus. However, one recent Nordic study has challenged our findings. They were investigating the impact of Nordic health inequality policies. Rather than focusing on specific NCDs, they aimed to investigate urban–rural variation in mortality across four Nordic countries by measuring life expectancy and potential years of life lost [83]. Findings indicate that, in three of the four countries, low population density was consistently associated with higher mortality rates. They surmise that this could be owing to a shift from industrial to service sectors in cities, and to lack of support from the welfare state for residents of rural areas in the Nordic context.

Nasca et al. (1980) had observed similar findings to this present study for all-cause and specific cancers [21]. A significant linear relationship had been observed for high population density and incidence of head and neck, oesophageal, lung, stomach and colorectal cancer for men and women. In women they observed a relationship with breast cancer and a U-shaped pattern for cervical cancer. In men only, they observed an association between pancreas, bladder and liver cancer. They posit that occupational exposures may be causing these relationships in men. Since 1990, the number of people dying from cancer has doubled, rising to 10 million across the globe [84]. In 2019, Western Europe had one of the highest number of cancer deaths across the globe (1.27 million) and the highest incidences along with North America and East Asia. Non-malignant and malignant melanoma skin cancers made up 70% of the total incidences. Greater attention is required by researchers, policymakers and planners in order to consider the implications of density on human health and to investigate the mechanisms underlying our findings.

Even though population density appears to have a low profile within the discipline of public health, the impact of population density on air pollution is gaining greater attention in other disciplines, such as economics [85]. One economic study used German panel data from 2002–2015 in order to estimate the effects of population density on air pollution [85]. As well as densely populated areas being more likely to experience violation of air pollutant thresholds, they found densely populated areas are more polluted, with emissions being derived from total residential energy use and commuting rather than emissions from industry. One study in this review revealed that mortality risks were associated with transport network patterns and population density. The study reported that mortality risk was 16% higher for women across cities with the highest junction density compared to the lowest third, and 12% higher for men. Standardised mortality ratios for premature mortality were higher in cities with higher minor road and junction density and percentage of population living within 100 metres of major roads for both sexes. Regression analyses revealed a statistically significant increase in all-cause and CVD and stroke premature mortality with increasing population density and transport-related metrics [46].

One of the earliest studies in this review was a North American large-scale study that included a latency period of 44 years in their ecological study of bladder cancer [39]. Bladder cancer rates increased as population density increased across quartiles. Mean population density of the highest quartile was 10 times higher than lowest. Based on a 1990 map of volatile organic compounds (VOCs), authors surmise that this association may reflect a growing number of vehicles and emissions during the latency period. A previous study outside this review evaluated the impacts of atmospheric emissions on human health in one Portuguese urban area [16]. They estimated human exposure as well as morbidity and mortality rates. Areas of high anthropic activity were associated with high levels of air pollution and usually found in urban centres. Their evaluation of abatement measures predicted a reduction in PM_10_ emissions by 8% and potential health benefits strongly correlated with high population density. This is important, as findings from a semi-individual cohort study, with a sample of 1.9 million cancer-free cases from Saxony, found that an increase of ambient PM_10_ by 10 mµ/m^3^ was associated with a 53% increase in relative risk of mouth and throat cancer and 52% increase in relative risk of non-melanoma skin cancer. Prostate and breast cancer were also modestly associated [86]. A recent longitudinal study conducted in Germany revealed a strong linear relationship between PM_10_ and PM_2.5_ quartile groups and event rates of hypoglycaemia and hypoglycaemic coma in children and adolescents [87].

In this review, a prospective cohort study, focusing on one Dutch city, found that population density was modestly associated with higher all-cause mortality and with positive and negative self-reported health outcomes such as smoking rates, urban stress and active transport [32]. Active travel was associated with a reduction in mortality, leading the researchers to question whether it is possible to mitigate against the negative effects of high population density in a compact city.

IS Global states that innovative and holistic strategies by urban and transport planners, such as air and noise abatement, could lead to a 20% reduction in premature deaths [7]. Urban designers are recommending that high-density housing be built away from heavily trafficked roads [88]. Based on our findings, further research should investigate if and when the advantages of high density outweighs the disadvantages—is there a tipping point that endangers public health and can be avoided by better planning and abatement measures? The amount and nature of green space and making active travel possible are important issues for planners of the built environment who must consider the implications of our findings. In addition, prospective parents and young people ought to be made aware of risk factors for T1D if living in areas of low population density, whether in urban or rural areas. Future research is required to investigate the relationship between population density and diabetes, and in particular to explore the effect of prevention strategies at an individual, group and service level.

### 6.1. Study Limitations

We conducted a comprehensive cross-disciplinary search of 10 databases; however, some studies may be undetected. Owing to the heterogeneity of statistical methods amongst the studies, only general trends are presented. These patterns are observations rather than evidence of causation. The majority of studies used a large or very large sample size. Measuring population density at large scale such as nations or counties could mask large, urbanised areas. Any significant findings could be due to the very high statistical power and narrow confidence intervals [89]. For ecological studies that use small area geographies, there is a risk of internal variability and hidden rare disease. Included studies varied in categorisation of population density, such as tertiles, deciles, mean per district, etc., influencing effect size and degree of granulation. All studies were ecological or included aggregate level data. Many ecological studies failed to state study design or attempt to justify the ecological design or choice of ecological unit, indicating a lack of standardised reporting. Some of the studies appear in various groups/subgroups because they report on a wide range of diseases. For example, five studies reported an association between population density and breast cancer, but none focused solely on breast cancer. Even though the modified quality assessment tool was an effective way to critique ecological studies, some criteria were less relevant for multi-level studies, such as reporting for cross level bias. Adopting an additional assessment tool may have strengthened quality assessment.

### 6.2. Conclusions

This is the first study, to the authors’ knowledge, to review the evidence regarding a relationship between population density and morbidity and mortality rates of NCDs. In order to tackle increasing rates of NCDs within the Western hemisphere, it is important to investigate each disease identified further at a local level, taking individual as well as area level variables into account. Further epidemiological studies would be desirable. Public health practitioners and planners need to take action to protect citizens from exposure to man-made emissions in order to reduce incidence and mortality rates from the NCDs highlighted in this review. The public need to be made aware of multiple interactions across space and population density and opportunities for protection and prevention. In areas of low density, health education and health promotion are required to raise awareness of risk and increase health protection.

## Supplementary Materials

The following are available online at https://www.mdpi.com/article/10.3390/ijerph19052638/s1, File 1: Data Extraction, Table S1: Example of search strategy, Table S2: Study Characteristics, Table S3: Critical Appraisal of Selected Studies; Table S4: Summary of Results.
