# Supplementary material for: Is Population Density Associated with Non-Communicable Disease in Western Developed Countries? A Systematic Review
Source: Int J Environ Res Public Health. 2022 Feb 24;19(5):2638. doi: 10.3390/ijerph19052638 (PMC8910328; doi:10.3390/ijerph19052638)
Supplement: Supplementary file 1 [file ijerph-19-02638-s001.zip › ijerph-1503496-supplementary/Table S1 Example of search strategy.pdf]

Table S1: Medline (EBSCO) search strategy June 2019

1	(MH "Population Density") or TI ("population densit*" or "urban densit*" or "residential densit*") or AB ("population densit*" or "urban densit*" or "residential densit*")	30,40
2	(MH "Crowding")	3,074
3	S1 OR S2	33,286
4	(MH "Morbidity+")	516,678
5	(MH "Mortality+")	359,791
6	(MH "Neoplasms+") or cancer* or oncol*	3,953,319
7	(MH "Cardiovascular Diseases+")	2,275,503
8	(MH "Endocrine System Diseases+")	937,453
9	(MH "Lung Diseases, Obstructive+")	202,368
10	(MH "Chronic Disease+")	255,715
11	S4 or S5 or S6 OR S7 OR S8 OR S9 OR S10	7,320,546
12	(MH "Health Status") OR (MH "Health Status Disparities")	89,732
13	(MH "Residence Characteristics")	31,413
14	TI ("census tract*") or AB ("census tract*")	2,479
15	(MH "Socioeconomic Factors")	147,284
16	(MH "Survival Analysis")	127,359
17	S12 OR S13 OR S14 OR S15 OR S16	373,560
18	S3 AND S11	3,386
19	S17 AND S18	615
20	S17 AND S18<br>Limiters - Date of Publication: 20000101-20191231	414
