# Supplementary material for: Is Population Density Associated with Non-Communicable Disease in Western Developed Countries? A Systematic Review
Source: Int J Environ Res Public Health. 2022 Feb 24;19(5):2638. doi: 10.3390/ijerph19052638 (PMC8910328; doi:10.3390/ijerph19052638)
Supplement: Supplementary file 1 [file ijerph-19-02638-s001.zip › ijerph-1503496-supplementary/Table S2 Study characteristics.pdf]

Table S2 Study Characteristics

**Year of publication**		
1991-2000	50, 54,72,76	4
2001-2010	28,35,36,42,43,44,11,47,49,51,53,55,56,57, 69,77	16
2011-2020	4,29,30, 31, 32,33,34,37,3,38,39,40,41,45,46,6,48,52,58, 59, 60, 61, 62, 63, 64, 65, 66, 67, 68, 70, 71, 73, 74, 75	34
**Study design**		
Ecological	3, 4,28,29,30,34, 39, 41,42,43,46,11,48,49,53,54,55,56, 57,58,62,63,64,65,66,67,68,69,70,71,72,73,75, 76, 77	35
Longitudinal & Cohort studies	31,32,33,35,36,37,38,40,44,45,50,51,59,60,61,74	16
Case control	52	1
Data-linkage study	6	1
Not explicit	47	1
**Adjustment for SES factors**		
Yes: Area level SES only	6,11, 28, 29, 30, 31, 33, 34, 38, 41,42,44,45,46, 48, 49, 53, 55,56,57,58,62,63,64,66,67,68, 70, 71,72,73,74,75,76	34
Yes: Area level SES AND Individual SES	32,35,36,37,40,52,59,60,61,77	10
No: neither area level SES nor individual SES	3,4,39,43,47,50,51,54,65,69	10
**Age range**		
Children/Adolescent	11, 30, 31, 42,43, 49, 51,52,53,55,56,57,58,64,67,71,72	17
Adults	3,4, 6,28,29,32,33, 34,35,36,37, 38,39,40,41,44,45,46, 47,48,50,54,59,60,61,62,63,65,66, 68,69,70,73,74,75,76,77	37
**Geographical Scope**		
Country	11,34,37,38,43,46, 47,51,59,60, 62, 65,66,67,70,71,73,74,75,76,77	21
State	6,28,29,30,33,42,44, 50,52,54,64,68,77	13
County	31,39,41,48,56,57,72	7
City	53	1
Region	4,35,36,40,49,55,58,61,69	9
Neighbourhood	32	1
Province	3	1
District	45	1
**Level of analysis**		
County	39, 43,44,47,48,50,52,62,68,77	10
Electoral district	34,37,45,51,69,71,74	7
Electoral/census ward	55,56,57,58,61,70,72	7
Census tract	28, 40,41,64	4
Census block group	6,29,33,40	4
Municipality	4, 11,38, 75	4
Neighbourhood	31,32	2
Parish	35,59,60	3
Local area	36,66	2

Region	49,67	2
Health district	30	1
Province	76	1
Administrative unit	3	1
City	46	1
Health service catchment area	53	1
PD quintiles (villages, towns, cities)	54	1
Local authority	63	1
Zip code tabulation areas (ZCTA	65	1
Parliamentary constituency	73	1

**Health Condition**		
Cancer	3,4,6,28,32,33,34,35,38,39,40, 47,50,54,55,56,57,58,60,61,68,70,75	23
Diabetes	11,30,41,42,43,45, 48,49,52,55,62,64,65,67,71,72,77	17
**Respiratory Diseases**		
Lung cancer	4,35,38,50,54,60,70	7
COPD	35	1
General Lung disease	32,77	2
Asthma	31,53,77	3
**Cardiovascular disease**	29,32,35,36,37,46,63,77	8
**Neurological Conditions**		
Alzheimer’s	47	1
Amyotrophic lateral sclerosis	66,69	2
**Congenital**		
Clubfoot	51	1
