# Supplementary material for: Is Population Density Associated with Non-Communicable Disease in Western Developed Countries? A Systematic Review
Source: Int J Environ Res Public Health. 2022 Feb 24;19(5):2638. doi: 10.3390/ijerph19052638 (PMC8910328; doi:10.3390/ijerph19052638)
Supplement: Supplementary file 1 [file ijerph-19-02638-s001.zip › ijerph-1503496-supplementary/Table S3 Critical appraisal of selected studies.pdf]

Table S3: Critical appraisal of selected studies. Modified scale from Dufault and Klar by Cortes-Ramirez et al. 2018

Author (year)	Study design and focus			Statistical methodology				Quality of reporting			Score points
	Sample size	Level of inference	Pre-specification of ecological units	Validity of statistical inference	Use of covariates	Spatial effects	Proper adjustment for covariates	Statement of study design	Justification of study design	Discussion of cross-level bias and limitations	
Agovino et al. (2018) [4]	2	1	1	2	1	1	0	1	1	0	10
Altekruse et al. (2010) [28]	2	1	1	2	1	1	0	1	1	1	11
Balamurugan et al. (2013) [29]	2	1	1	2	1	1	1	0	0	0	9
Ball et al. (2014) [30]	2	1	0	0	1	1	0	1	0	0	6
Beck et al. (2013) [31]	2	1	1	1	1	0	0	1	1	1	9
Beenackers et al. (2018) [32]	2	1	1	2	1	0	1	1	1	1	11
Canchola et al. (2017) [33]	2	1	1	2	1	0	1	1	0	0	9
Carsin et al. (2011) [34]	2	1	1	2	1	1	1	0	0	1	10
Chaix et al. (2006) [35]	2	1	1	2	1	1	1	1	1	1	12
Chaix et al. (2007) [36]	2	1	1	2	1	0	1	1	1	0	10
Chandrabose et al. (2009) [37]	1	1	1	0	1	0	1	1	1	1	8
Chawińska et al. (2014) [3]	2	1	0	2	0	1	0	0	1	0	7

Chouaid et al. (2017) [38]	2	1	1	2	1	0	1	1	1	1	11
Colli et al. (2012) [39]	1	1	1	2	0	1	0	0	1	0	7
DeRouen et al. (2018)[40]	2	1	0	2	1	0	1	1	0	0	8
Drewnowski et al. (2014) [41]	2	1	1	2	1	1	1	1	1	1	12
Du Prel et al. (2007) [42]	2	1	0	0	1	1	1	1	0	1	8
Elliott et al. (2010) [43]	2	1	1	2	1	0	1	0	1	1	10
Erwin et al. (2010) [44]	2	1	0	0	1	0	0	1	1	0	6
Faka et al. (2009) [45]	2	1	1	0	1	1	0	1	1	0	8
Fecht et al. (2016) [46]	2	1	1	0	1	0	1	1	1	1	9
Gomez et al. (2011) [6]	2	1	1	2	0	0	0	0	1	0	7
Gopinath et al. (2008) [11]	1	1	0	0	1	0	1	0	0	1	5
Hallberg et al. (2007) [47]	2	1	0	2	0	0	0	0	0	0	5
Hipp (2015) [48]	2	1	0	2	1	1	0	1	1	0	9
Holmqvist et al. (2008) [49]	2	1	1	2	1	0	1	0	0	1	9
Howe et al. (1993) [50]	2	1	0	2	1	0	0	0	1	1	8
Krogsgaard et al. (2006) [51]	2	1	1	2	0	0	0	1	1	0	8
Liese et al. (2018) [52]	1	1	0	2	1	0	1	0	0	0	6

Lovasi et al. (2008) [53]	2	1	0	0	1	0	1	1	0	1	7
Mahoney (1990) [54]	2	1	1	2	1	0	0	1	1	1	10
Manda et al. (2009) [55]	2	1	0	2	1	1	0	1	1	0	9
McInally et al. (2003) Leukaemia [56]	2	1	0	2	0	0	0	1	0	1	7
McInally et al. (2003) Wilms tumour [57]	2	1	0	2	1	1	0	1	0	1	9
McInally et al. (2015) [58]	2	1	0	2	1	0	1	1	0	1	9
Meijer et al. (2012) [59]	2	1	1	2	1	0	1	1	1	1	11
Meijer et al. (2013) [60]	2	1	1	2	1	0	1	0	1	0	9
Muquit et al. (2015) [61]	2	1	0	2	0	0	0	1	0	0	6
Nguyen et al. (2019) [62]	1	1	0	2	1	1	1	0	0	0	7
Phillips et al. (2017) [63]	2	1	0	1	1	0	0	1	0	0	6
Puett et al. (2012) [64]	2	1	0	2	1	0	1	1	0	1	9
Rogers (2019) [65]	2	1	0	2	1	0	0	0	0	0	6
Rooney et al. (2015) [66]	2	1	1	2	1	1	1	1	1	0	11
Samuellson et al. (2020) [67]	2	1	0	0	0	1	0	0	0	0	4

Schwartz et al. (2019) [68]	2	1	0	0	1	0	1	1	1	1	8
Scott et al. (2010) [69]	2	1	0	2	0	0	0	0	0	0	5
Sharp et al. (2014) [70]	2	1	0	2	1	0	1	1	0	1	9
Sheehan et al. (2020) [71]	2	1	1	2	1	1	1	1	1	1	12
Staines et al. (1997) [72]	2	1	1	2	1	0	1	1	0	0	9
Tunstall et al. (2012) [73]	2	1	1	2	1	0	0	0	0	0	7
Van Cauwenberg et al. (2019) [74]	2	1	1	2	1	0	0	1	1	0	9
Van der Aa et al. (2011) [75]	2	1	0	2	1	0	0	0	0	1	7
Villanueva et al., (2000) [76]	2	1	0	0	1	0	0	1	1	1	7
Wickrama et al. (2005) [77]	2	1	0	0	1	0	1	0	0	0	5
