# Supplementary material for: Is Population Density Associated with Non-Communicable Disease in Western Developed Countries? A Systematic Review
Source: Int J Environ Res Public Health. 2022 Feb 24;19(5):2638. doi: 10.3390/ijerph19052638 (PMC8910328; doi:10.3390/ijerph19052638)
Supplement: Supplementary file 1 [file ijerph-19-02638-s001.zip › ijerph-1503496-supplementary/Table S4 Summary of results.pdf]

Table S4 Summary of Results

Reference	**Author**	**Results**			**Remarks**
4	Agovino et al.,(2018)	**Spatial Durbin model estimates**			Population Density (People/km^2)
		Variables	Direct effects	Spatial spillover effects	
		Stomach Cancer MR	0.0001**(SE:0.00007)	-0.00004(SE:0.0001)	
		Colon Cancer MR	0.0002**(SE:0.00009)	0.0005***(SE:0.0001)	
		Liver Cancer MR	0.0003**(SE:0.00006)	0.0007***(SE:0.0001)	
		Pancreatic Cancer MR	0.0003**(SE:0.00006)	-7.75E-6(SE:0.00008)	
		Lung Cancer MR	-0.00009(SE:0.0001)	0.001***(SE:0.0001)	
		Breast cancer MR	0.0002***(SE:0.00006)	0.0006***(SE:0.0001)	
		Female Genital Cancer MR	0.0001**(SE:0.00007)	0.0001(SE:0.0001)	
		Male genital Cancer MR	0.0001**(SE:0.00007)	-0.0001(SE:0.0001)	
		Urinary Tract Infection MR	0.00009(SE:0.00007)	0.0003***(SE:0.0001)	
		Leukemia MR	0.0005***(SE:0.00006)	0.0004***(SE:0.0001)	
		***, **, * indicate statistical significance at the 1%, 5% and 10% levels, respectively.			
28	Alterkruse et al.,(2010)	% of Black population	Population Density	RR (highest recorded Cluster)	
		Black pop <2.5	Urban	8.2	
			Sub-Urban	1.5	
		Black pop >2.5	Urban	2.3	
			Sub-Urban	5.5	
29	Balamurugan et al.,2013	Analysis showed that 4.5% to 9% of the deviance in stroke mortality between BGs can be explained by poverty, education, **population density**, and population mobility			
30	Ball et al.,(2014)		Incidence rate ratio of Type1 DM per 100people /km^2		
		Unadjusted Fixed effect model	1.004(0.996-1.011)		
		Adjusted Fixed effect model	1.001(0.987-1.015)		

		Unadjusted mixed model(Fixed+ random effect)	1.008(0.992-1.029)	
31	Beck et al.,(2013)	Correlation coefficient of population density (persons/$mile^2$): r=0.035;p=0.0008		
32	Beenackers et al.,(2018)	Association Between population density and All –Cause Mortality (29.88%)		Population density for the regression analysis was measured in 10 persons per hectares
		HR:1.048(1.026-1.070) p<0.0001		
		HR:1.037(1.016- 1.058, p< 0.0001		Adjusted for individual education level
		HR: 1.026(1.007- 1.050, p<0.0001		Adjusted for Neighborhood Educational Level
		**Cause-specific Mortality**		
		Cancer mortality (10.21%)		
		HR:1.043(1.011-1.076); p=0.008		
		HR:1.036(1.003- 1.069);p< 0.030		Adjusted for individual education level
		HR: 1.033(0.999- 1.068);p=0.059		Adjusted for Neighborhood Educational Level
		Cardiovascular Mortality (9.15%)		
		HR:1.053(1.014-1.093); p=0.006		
		HR:1.041(1.003- 1.080);p< 0.036		Adjusted for individual education level

		HR: 1.029(0.989- 1.070);p=0.152			Adjusted for Neighborhood Educational Level
		Respiratory Mortality (3.14%)			
		HR:1.072(1.000-1.149); p=0.049			
		HR:1.056(0.098- 1.132);p< 0.126			Adjusted for individual education level
		HR: 1.041(0.967- 1.121);p=0.282			Adjusted for Neighborhood Educational Level
33	Canchola et al,.(2017)		HR for Colorectal cancer Risk		
			Males	Females	
		Q5 high(ref)			
		Q4	1.02(0.82-1.28)	0.97(0.78-1.20)	Statistically non-significant HRs
		Q3	0.99(0.78-1.27)	0.97(0.77-1.24)	
		Q2	1.05(0.81-1.37)	1.00(0.77-1.30)	
		Q1 low	0.97(0.72-1.31)	0.91(0.68-1.21)	
34	Carsin et al.,(2010)	Population Density	Males	Females	
			All Non-melanoma Cancer		
		Rural(ref)			
		Intermediate	1.11(1.05-1.16)	1.15(1.09-1.21)	
		Urban	1.19(1.14-1.24)	1.39(1.33-1.45)	
		P trend	<0.001	<0.001	
			Basal cell Carcinoma		
		Rural (ref)			
		Intermediate	1.20(1.13-1.27)	1.19(1.12-1.26)	
		Urban	1.35(1.28-1.42)	1.48(1.40-1.55)	
		P trend	<0.001	<0.001	
			Squamous Cell Carcinoma		
		Rural (ref)			
		Intermediate	0.96(0.89-1.03)	1.19(0.95-1.15)	
		Urban	0.94(0.88-1.01)	1.16(1.07-1.26)	

		P trend		0.085			0.004	
35	Chaix et al.,(2006)	Study did not resolve a specific quantitative outcome to describe the dose–response association between population density and mortality from lung cancer and COPD in all age groups investigated, and from IHD especially in the youngest age group (after adjustment for individual and contextual socioeconomic status) Overall, the population density effect was the strongest on lung cancer mortality.<br>Additional impact only in 2 out of 16 subgroups of age.						
36	Chaix et al.,(2007)	No quantitative measure of direct effect of Population density, however in models stratified by population density, it was observed that the socio-economic contextual effect on ischemic heart disease (IHD) mortality tended to increase with population density.						Area population density was computed as the number of 50–79-year-old residents/km^2
37	Chandrabose et al.,(2019).	After adjusting for baseline population density, a 1% annual increase in population density was associated with smaller increases in<br>Waist Circumference (b= -0.047 cm/y; 95% CI, -0.067 to -0.026 [P<0.001]),<br>Weight (b= -0.025 kg/y; 95% CI, -0.044 to -0.006 [P=0.01]),<br>HDL-C (b= -0.038 mg/dL per year; 95% CI, -0.067 to -0.009 [P=0.009]).						
3	Chawińska et al.,2014	An increase in population density of 1000 /km^2 results in 13% growth in risk of cancers in men and 16% increase in this risk in women.						
38	Chouaïd et al.,(2017)							
		Non-Metastatic Disease At Diagnosis [HR,95%CI]			Metastatic Disease At Diagnosis [HR,95%CI]			
		One year Mortality			One year Mortality			
			Unadjusted	Adjusted		Unadjusted	Adjusted	
		Population density			Population density			
		Very low	1.14(1.06-1.23)**	1.06(0.98-1.15)	Very low	1.02(0.96-1.07)	0.96(0.90-1.02)	** =statistically significant
		Low	0.99(0.91-1.06)	0.95(0.88-1.03)	Low	0.98(0.93-1.04)	0.97(0.92-1.03)	
		High	1.05(0.97-1.13)	1.02(0.94-1.10)	High	0.98(0.93-1.04)	0.96(0.91-1.02)	
		Very High (ref)			Very High (ref)			
		Two year Mortality			Two year Mortality			

		Very low	1.02(0.96-1.07)	0.96(0.90-1.02)	Very low	1.01(0.96-1.06)	0.95(0.90-1.01)	
		Low	0.98(0.93-1.04)	0.97(0.92-1.03)	Low	1.00(0.95-1.05)	0.99(0.94-1.05)	
		High	0.98(0.93-1.04)	0.96(0.91-1.02)	High	0.98(0.93-1.03)	0.95(0.90-1.00)	
		Very High (ref)			Very High (ref)			
39	Colli et al.,(2012)	Correlation coefficients (R) between bladder cancer mortality rates and the population densities were r = 0.37, P<0.001 for men and r = 0.28, p<0.001 for women. In addition, population densities increased with increasing bladder cancer mortality rates across all quartiles.						
40	DeRouen al.,(2018)							
		Population density(quartiles)		Localized Prostate Cancer (OR,95%CI)			Advanced Prostate Cancer (OR,95%CI)	
		Q_1 (Ref)						
		Q_2		0.63(0.38-1.04)			0.76(0.52-1.12)	
		Q_3		0.49(0.30-0.81)			0.74(0.51-1.09)	
		Q_4		0.41(0.25-0.67)* [p<0.01]			0.56(0.39-0.82)* [p<0.01]	
41	Drewnowski et al.,2014	In bivariate analyses, 24% of the variance in diabetes prevalence was explained by median home value. Adding population density, race/ethnicity, age, and spatial dependence to the model increased the variance explained to 48%.						
42	du Prel et al.,(2007)							a=Models including sex, age and each socioeconomic indicator separately
					Simple Poisson regression[a]		Multiple Poisson regression[b]	b= Model including sex, age and all socioeconomic indicators with risk estimates presented in this column
		Relative risk (RR) per increase of 1,700 persons per km^2			RR: 0.878(95%CI: 0.813-0.948);p<0.001		RR: 0.961(95%CI: 0.834-1.106);p=0.576	

43	Elliott et al.,(2010)	Adjusting for age, there was a significant positive association between Type 1 Diabetes incidence and the proportion urban: RR = 1.19 (95% CI 1.05–1.34), p = 0.004.; as at high population densities type 1 diabetes incidence increased with increasing ambient Ultra Violet Ray.	
44	Erwin et al.,(2010)	Spearman Rank Correlation between SMR and population density :rho: 0.4028; p=0.0149	(for 36 countries)
		Multivariate Regression Analysis: β =0.0001770, t value =2.18, p=0.037	
		Spearman Rank Correlation between SMR and population density: rho: 0.4742; p=0.0013	(in 43 units)
		Multivariate Regression Analysis: β =0.0000488, t value =0.33, p=0.743	
45	Faka et al.,(2018)	The beta coefficient of population density as a factor of five random effect levels according to population density intervals in ascending order (i.e. from less densely populated to most densely populated) in quasi-Poisson regression analysis =1.81	
46	Fecht et al,(2016)	**Association between Population density and All-cause mortality**	
		Model 1 (Adjusted for age)	
		Females (<65 years)	*Significant at (p<0.05)
		T2 : RR: 1.09[1.07- 1.11]*	Population density was categorized into tertiles. RR was calculated with the lowest tertile as the reference group
		T3 **:** RR: 1.17 [1.15-1.19]*	(T2):2nd tertile and (T3) :3rd tertile
		Males (<65 years)	
		T2: RR : 1.12 [1.10- 1.14]*	
		T3: RR : 1.19 [1.18 -1.21]*	
		**Model 2 (additionally adjusted for deprivation and lung cancer mortality)**	
		Females (<65 years)	
		T2 : RR: 1.02 [0.99 – 1.04]	
		T3 **:** RR: 1.10 [1.09 – 1.13]*	
		Males (<65 years)	

		T2: RR:1.01 [0.99 – 1.02]			
		T3:RR :1.09 [1.07 – 1.10]*			
		Cardiovascular mortality			
		**Model 1 (Adjusted for age)**			
		Females (<65 years)			
		T2 : RR :1.15 [1.10 – 1.21]*			
		T3 : RR: 1.22 [1.16 – 1.27]*			
		Males (<65 years)			
		T2: RR :1.17 [1.14 – 1.21]*			
		T3: RR : 1.21 [1.17 – 1.24]*			
		**Model 2 (additionally adjusted for deprivation and lung cancer mortality)**			
		Females (<65 years)			
		T2 : RR: 1.04 [0.99 – 1.09]			
		T3 : RR: 1.13 [1.07 – 1.18]*			
		Males (<65 years)			
		T2: RR: 1.04 [1.01 – 1.07]*			
		T3: 1. 10 [1.06 – 1.13]*			
		Pearson Correlation coefficient: r =0.324 (between SMR All-cause in males <65 years and population/km^2			
6	Gomez et al., (2007).	No statistical association parameter was presented. Study indicated that Black colorectal cancer patients tended to live in neighborhoods with higher population density, shorter block length and size, and more walkable destinations.			
11	Gopinath et al.,(2008)	SIR by level of Population density: High [22.11]; Intermediate high[25.29];intermediate low[21.80]; Low[34.02]; chi square =22.733; p<0.0001, i.e. Areas of low population density had higher incidence rates of Type 1 Diabetes compared to those of high population density.			
47	Hallberg et al.,(2007)	Correlation coefficient between population density and Prostate cancer(r^2=0.1317,p=0.106); Alzheimer's (r^2=0.2494,p=0.021); Sick days in 2002 (r^2=0.6574,p<0.0001); Long term sick per 1000 persons (r^2=0.5331,p<0.0001); Leukemia ((r^2=0.0111,p=0.05)			
48	Hipp et al.,2015	Parameters of Population density as a factor in OLS model explaining 58.8% of county-level prevalence of diabetes (β =0.000192;t value=8.93,p=0.0001)			
49	Holmqvist et al.,(2008).	IRR for Type 1 diabetes in relation to Population density at the time of birth of children who later developed Type 1 diabetes with Poisson regression model			
		Population density	Unadjusted IRR	Adjusted IRR	
		>5000 persons	1.27(1.09-1.47); p=0.01	1.23(1.04-1.45);	
		<5000 persons	1.31(1.13-1.50)	1.28(1.1-1.50)	
		Rural areas (Ref)			

50	Howe et al.,(1993)	In black females, all cancers combined and cancers of the breast, cervix, and nervous system showed monotonic increases with statistically significant trends (p<0.05)				
		In white females, monotonic increases in cancer rate, significant at the 0.001 level, were noted for all cancers combined; cancers of the oral cavity, esophagus, stomach, liver, pancreas, breast, and ovary; and for Hodgkin's disease, non-Hodgkin's lymphomas, and the leukemias. Cancer rates for lung, corpus uteri, bladder, and nervous system increased across population density groups I (Rural), II(Small Urban), and III(Sub-urban), while the rates in group IV(Urban) were similar to or less than those in group III. This pattern of increase was statistically significant at the 0.001 level.				
		Among white males, a monotonic increase in the cancer rate, with statistical significance at the 0.001 level, was noted for cancers of the esophagus, stomach, colon, liver, pancreas, bone, testis, bladder, and nervous system; Hodgkin's disease and non-Hodgkin's lymphomas; and the leukemias				
		Among black males, the rates increased monotonically only for liver cancer and Hodgkin's disease. Only the trend in liver cancer was statistically significantly associated with population density (p<0.001)				
51	Krogsgaard et al.,(2006).	The incidences of isolated and combined Club Foot (CF) in the 30 districts of Denmark with the highest population density (more than 500 inhabitants/km^2) was 1.35. The calculated standardized morbidity ratio was significantly increased to 1.12 (CI 1.02–1.23) for isolated CF, and reduced to 0.84 (CI 0.65–1.08) for combined CF in these communities (not significant). There was a significant increase in population density with increasing incidence of CF in both sexes (p < 0.001).				
52	Liese et al.,(2018)					
			Unadjusted[a]	Adjusted[b]	Adjusted[c]	
		<500 resident/mile^2	2.81(1.62-4.90)*	2.24(1.10-4.58)*	3.22(1.40-7.41)	
		500-999 resident/mile^2	3.23(1.23-8.76)*	2.28(0.74-7.02)	3.36(0.96-11.8)	
		1000+ resident/ mile^2 (Ref)				
		a:Unadjusted: neighborhood characteristics alone.				
		b:Adjustment[1]: Age, gender, race/ethnicity, study site.				
		c:Adjustment[2]: Age, gender, race/ethnicity, study site, and breastfeeding, mother's diabetes, parental education , parental income.				
53	Lovasi et al.,(2008)	Correlation between Population density and hospitalization as a result of Asthma in children : -0.29				
54	Mahoney et al.,(1990)	In males there was a significant, direct linear relationship between increasing population density and all cancer sites combined, and for cancers of the oral cavity and pharynx, esophagus, stomach, colon, rectum, liver, pancreas, larynx, lung, bladder, brain, and nervous system, and for Hodgkin's disease and multiple myeloma (p<0.05)<br>Among females, a significant, direct linear relationship was observed between increasing population density and all cancer sites combined, and for cancers of the buccal cavity and pharynx, esophagus, stomach, lung, breast, and				

<table>
<tr><td></td><td></td><td colspan="4">kidney. Malignant melanomas of the skin, and in situ and invasive cancers of the cervix exhibited unusual incidence patterns across the population density quintiles.</td><td></td></tr>
<tr><td>55</td><td>Manda et al.,(2009)</td><td colspan="4">Correlation between Population density and Type 1 diabetes : -0.006.</td><td></td></tr>
<tr><td>56</td><td>McNally et al., (2003).</td><td colspan="4">Ecological analyses showed that there was a significant monotonic relationship between acute lymphoblastic leukaemia (ALL) incidence and population density (p <0.05). Higher rates were seen in the more densely populated wards with: Q_2 vs Q_1:RR of 1.11(0.84-1.46);Q_3 vs Q_1:RR of 1.20(0.92-1.57);Q_4 vs Q_1:RR of 1.21(0.92-1.58);Q_5 vs Q_1:RR of 1.30(0.99-1.69)</td><td></td></tr>
<tr><td>57</td><td>McNally et al., (2003).</td><td colspan="4">There was a non-linear relationship between Wilm's Tumour incidence and population density (P=0.008), as highest incidence rates were found in both the least densely populated quintile and the most densely populated quintile. There was no statistically significant association between Soft tissue sarcomas and Population density.</td><td></td></tr>
<tr><td>58</td><td>McNally et al.,(2015)</td><td colspan="4">Univariate Cox regression models indicated that population density did not affect the risk of death (P =0.522) from non–seminoma testicular cancer.</td><td></td></tr>
<tr><td>59</td><td>Meijer et al.,(2012)</td><td colspan="4">Individuals between 30 and 49 years living in areas with the lowest population density had a significantly lower mortality (HR:0.85, CI: 0.76–0.95) than those living in more populated areas.</td><td></td></tr>
<tr><td></td><td></td><td colspan="4">Residents between the age of 50 and 64 years living in the least populated areas had lower mortality (HR:0.81, CI:0.76–0.86) compared to those living in most densely populated areas. The oldest age group had lower mortality in the least populated areas (HR:0.86, CI:0.83–0.89) compared to those living in most densely populated areas.</td><td></td></tr>
<tr><td></td><td></td><td>Population density</td><td>Ages 30-39</td><td>Ages 50-64</td><td>Ages 65-81</td><td></td></tr>
<tr><td></td><td></td><td>Highest (ref)</td><td colspan="3">Hazard ratio and 95%CI of individual factors on individual all-cause mortality</td><td></td></tr>
<tr><td></td><td></td><td>High</td><td>0.88(0.83-0.94)</td><td>0.91(0.87-0.94)</td><td>0.89(0.87-0.91)</td><td></td></tr>
<tr><td></td><td></td><td>Low</td><td>0.86(0.78-0.94)</td><td>0.83(0.79-0.88)</td><td>0.88(0.85-0.90)</td><td></td></tr>
<tr><td></td><td></td><td>Lowest</td><td>0.85(0.76-0.95)</td><td>0.81(0.76-0.86)</td><td>0.86(0.83-0.89)</td><td></td></tr>
<tr><td>60</td><td>Meijer et al.,(2013)</td><td colspan="4">A significantly lower HR of breast cancer was found in areas with low population density (HR:0.93; CI: 0.88 to 0.99); Risk of lung cancer was lower in areas with lowest population density (HR¼0.80; CI 0.74 to 0.85)</td><td></td></tr>
<tr><td></td><td></td><td>Population density</td><td>HR for
Breast Cancer Incidence</td><td colspan="2">HR for
Lung Cancer Incidence</td><td></td></tr>
<tr><td></td><td></td><td>Highest (ref)</td><td></td><td colspan="2"></td><td></td></tr>
<tr><td></td><td></td><td>High</td><td>0.95(0.91-0.99)</td><td colspan="2">0.87(0.84-0.91)</td><td></td></tr>
<tr><td></td><td></td><td>Low</td><td>0.93(0.88-0.99)</td><td colspan="2">0.82(0.77-0.86)</td><td></td></tr>
<tr><td></td><td></td><td>Lowest</td><td>0.94(0.87-1.01)</td><td colspan="2">0.80(0.74-0.85)</td><td></td></tr>
<tr><td>61</td><td>Muquit et al., (2015)</td><td colspan="4">There was a Statistically significant correlation between lower population density and higher incidence of glioblastoma: -0.191,p=0.0015</td><td></td></tr>
</table>

62	Nguyen et al.,(2019)	Tertiles of population density were significantly related to health outcomes.				
			Prevalence difference (95%CI)			
		**Population density**	Obesity	Diabetes	Physical inactivity	
		1st Tertile	2.82(2.56-3.07)*	0.54(0.42-0.67)*	2.36(2.08-2.65)*	
		2nd Tertile	2.16(2.04-2.28)*	0.51(0.46-0.56)*	1.52(1.39-1.66)*	
63	Phillips et al., (2017)	There were strong relationships between domestic air pollution and chronic rheumatic heart disease (RHD). Inclusion of published data on social class, education, crowding and population density in multiple regression analyses showing that the air pollution association was independent of these; only overcrowding was separately linked with RHD				
64	Puett et al., (2012)	The study found overall that risk of T1DM incidence tended to increase for children living in increasingly affluent Census tracts, regardless of population density and individual-level race/ethnicity.				
		**Population density**	**SIR of T1DM**		RR (95%CI)	
		<500	0.93(0.85-1.02)		1.04(0.93-1.18)	
		500- <1000	1.15(0.99-1.33)		1.26(1.07-1.48)	
		≥1000	0.98(0.91-1.04)			
65	Rogers et al., (2019)	Low population density associated with diabetes RR: 2.28(2.08-2.50)				
66	Rooney et al., (2015)	Association between **Amyotrophic Lateral Sclerosis (ALS) rates and population density.**<br>Two clusters showed significant low frequency of ALS. But did not correlate with population density or residents' social deprivation<br>An elliptically shaped cluster consisting of 656 SA's (population: 152,508) located centered on the Kilkenny/Carlow border region included 25 cases when 61.9 were expected (RR=0.390; P=0.048)<br>Second cluster consisting of 271 SA's (population: 56,638) was located on the coastline of County Clare where 2 cases were observed instead of the 24 expected (RR=0.082; P=0.028).				
67	Samuelsson et al., (2020)	**Diabetes**<br>Densely populated Nordic areas, such as major cities and southern Sweden, had the lowest incidence, yet higher than most neighboring countries.<br><br>Incidence generally decreased with population density for all countries (negative Binomial regression coefficient for density: −1.4e−4 95% CI: [−2.2e−4, −6.0e−5], P < .001), and the effect of density did not differ between countries (P > .05)				
68	Schwartz et al. (2019)	**Colorectal Carcinoma (CRC)**				

		CRC incidence rates varied almost threefold across ND counties, from 29.2 in Renville County to 86.4 per 100,000/year in Oliver County.<br>Multiple linear regression: Population density (p = 0.081) and well water (p = 0.067) significantly predicted CRC rate with p values < 0.10, (using “forward selection” only in the regression model)	
69	Scott et al., (2010)	**ALS**<br>People with ALS were more likely to be resident in areas of high population density at diagnosis.<br>Population density in region of residence at diagnosis explained 25% of the variance in ALS rates (r = 0.5, p < 0.01).	
70	Sharp et al., (2014)	Risks were significantly higher in both sexes in urban than rural residents as follows:	

**Outcomes (Cancers)**	Males	Female	After adjusting for socioeconomic variation, urban-rural differences were evident for 12 of 18 cancers
Head and neck	RR urban vs. rural = 1.53, 95 % CI 1.42-1.64	RR = 1.29, 95 % CI 1.15-1.45	
esophageal	1.21 (1.11-1.31)	1.21 (1.08-1.35)	
stomach	1.36 (1.27-1.46)	1.19 (1.08-1.30)	
colorectal	1.14 (1.09-1.18)	1.04 (1.00-1.09)	
lung	1.54 (1.47-1.61)	1.74 (1.65-1.84)	
non-melanoma skin	1.13 (1.10-1.17)	1.23 (1.19-1.27)	
bladder	1.30 (1.21-1.39)	1.31 (1.17-1.46)	
	Prostate cancer risk was higher in rural areas (0.94, 0.90-0.97).	Risks of, cervical, kidney and brain cancer were significantly higher in females in urban areas	
*Other cancers showed no significant urban-rural differences			

71	Sheehan et al., (2020)	**Childhood Type 1 Diabetes**<br>An association with the living environment (RR 0.995; 95% CrI 0.991, 0.998) and radon potential class (RR 1.044; 95% CrI 1.015, 1.074), with 85% of the variability in type 1 diabetes risk explained by the spatial structured component.<br><br>Ecologic regression analysis included nitrogen dioxide (as a marker of air pollution), lead in soil, radon potential class, ethnicity, overcrowding and index of multiple deprivation (IMD) living environment domain.<br><br>With adjustment for these variables fewer local authority districts with an 80% probability of having a higher (74/354 vs 87/354) or lower (79/354 vs 88/354) risk compared with England as a whole.	

72	Staines et al., (1997)	**Childhood insulin-dependent diabetes**<br>Incidence rates were significantly lower in wards of higher population density and overcrowded houses<br>The RR for areas in the upper half of the childhood density distribution was 0.88 (95% confidence interval (CI): 0.78-0.99) and for the two upper tertiles of household overcrowding the rate ratios were 0.84 (95% CI: 0.74-0.95) and 0.68 (95% CI: 0.58-0.79) respectively.	
73	Tunstall et al., (2012)	**All-cause mortality**<br>A statistically significant negative association was found between overcrowding and death rates among the 7th–10th most deprived deciles of −0.327, −0.516, −0.263 and −0.368, respectively.<br><br>In a model where the association between death rates, Carstairs, population density, black and ethnic minorities (BME) and change, when all constituencies were considered, there was a statistically significant positive coefficient of 0.849 for Carstairs. Population density, BME and change had negative coefficients of −0.083, −0.311 and −0.166, respectively.	
74	Van Cauwenberg et al., (2019)	**Diabetes**<br>In lower SES neighborhoods, post-challenge plasma glucose increased by 6% in low-density, remained stable in medium-density and decreased by 3% in high-density neighborhoods. In medium SES neighborhoods, glucose remained stable in high-density, but increased by 2% and 3% in medium- and low-density neighborhoods, respectively.<br>In higher SES neighborhoods, no significant interaction effect between time and density was observed.	
75	Van der Aa et al., (2011)	**Melanoma**<br>The lowest SES-group had a significantly lower incidence than the highest SES-group; 10.2 (95% CI: 9.1-11.3) and 14.3 (95% CI: 12.9-15.8), respectively. Increased risk of melanoma was seen in municipalities with high population density, few people living on social security and many people with high income. Patients living in low SES neighbourhoods were diagnosed more often with higher stage disease (13% (95% CI: 12.3-13.8) diagnosed with pT4) than those living in high SES neighbourhoods (9% (95% CI: 8.5-9.8) diagnosed with pT4) ($p<0.001$) and with higher Breslow thickness ($p<0.001$).	
76	Villaneuva et al., (2000)	**Mortality**<br>There was an increase in convergence of macroeconomic indicators (**except for population density**), a lack of homogenisation of mortality rates and divergence of infant mortality rates (IMRs) during the study period	
77	Wickrama et al., (2005)	**Morbidity(Adverse physical health)**<br>After several incremental multilevel regression analysis, the percentage of farm families in counties was significantly associated with adverse physical health of adults, independent of individual and family characteristics, county poverty rate, ethnic composition, and population density. (all coefficients statistically significant at $P<0.01$ or <0.05)	
